# An all-silicon single-photon source by unconventional photon blockade

**DOI:** 10.1038/srep11223

**Published:** 2015-06-10

**Authors:** Hugo Flayac, Dario Gerace, Vincenzo Savona

**Affiliations:** 1 Institute of Theoretical Physics, Ecole Polytechnique Fédérale de Lausanne (EPFL), CH-1015 Lausanne, Switzerland; 2 Department of Physics, University of Pavia, I-27100 Pavia, Italy

## Abstract

The lack of suitable quantum emitters in silicon and silicon-based materials has prevented the realization of room temperature, compact, stable, and integrated sources of single photons in a scalable on-chip architecture, so far. Current approaches rely on exploiting the enhanced optical nonlinearity of silicon through light confinement or slow-light propagation, and are based on parametric processes that typically require substantial input energy and spatial footprint to reach a reasonable output yield. Here we propose an alternative all-silicon device that employs a different paradigm, namely the interplay between quantum interference and the third-order intrinsic nonlinearity in a system of two coupled optical cavities. This *unconventional photon blockade* allows to produce antibunched radiation at extremely low input powers. We demonstrate a reliable protocol to operate this mechanism under pulsed optical excitation, as required for device applications, thus implementing a true single-photon source. We finally propose a state-of-art implementation in a standard silicon-based photonic crystal integrated circuit that outperforms existing parametric devices either in input power or footprint area.

The last decade has witnessed a tremendous progress in silicon-on-insulator (SOI) technology for applications in photonic integrated computing and data processing^1^. In parallel, integrated photonic circuits have become increasingly appealing to realize key tasks in quantum information and communication, thanks to their natural interfacing with long distance communication networks working in telecommunication band (1.3–1.5 *μ*m wavelengths). Clearly, the combination of these two paradigms will likely allow to realize complex quantum operations on-chip that are far beyond what may be envisaged with table-top experiments, with significant and large scale impact on efficient and secure data processing and transmission^2^. Within this context, the generation of single photons plays a central role for the development of on-chip quantum photonic technologies^3^. In particular, the recent advances in silicon-based quantum photonics^4^,^5^,^6^,^7^ would strongly benefit from integrated single-photon sources on the same operating chip.

Single-photon sources on-demand can be realized with artificial two-level emitters, such as semiconductor quantum dots^8^,^9^, which have increasingly improved their radiative efficiency over the last few years^10^,^11^,^12^,^13^. However, these single photon sources are typically based on III-V semiconductors, they work most efficiently at cryogenic temperatures, and integration with silicon-based nanophotonic circuits working in the telecommunication band^1^ remains challenging. As a possible alternative, integrated single-photon sources in silicon-on-insulator (SOI) photonic circuits have been shown^14^,^15^,^16^, based on enhanced four-wave mixing induced by the silicon *χ*^(3)^ susceptibility and non-deterministic heralding. Even if the efficiency of such integrated sources can be improved by spatial multiplexing^17^, compactness and scalability remain open issues.

An alternative route to single-photon generation relies on the photon blockade mechanism^18^, where a strong third-order nonlinearity in an optical resonator enables a shift of the resonant frequency by more than its linewidth when a single photon is already present. As a consequence, the device can absorb a photon only after the previous one has been emitted. However, this mechanism requires a stronger optical nonlinearity than what is achieved in state-of-the-art SOI devices^19^.

Here, we build on the mechanism called *unconventional photon blockade* (UBP), recently advocated as a promising paradigm for single-photon generation^20^,^21^,^22^. The UPB mechanism relies on quantum interference, and is therefore highly sensitive to an optical nonlinearity of small magnitude. At difference with the conventional blockade, it has been shown that UPB can also occur when the nonlinear frequency shift per photon is much smaller than the cavity linewidth, which is usually the case also in silicon photonic crystal nanocavities^23^,^24^,^25^. So far, such theoretical mechanism was only shown to work under continuous-wave (cw) excitation, which severely limits the usefulness of the proposed antibunched radiation as an actual single-photon source^21^,^22^. In the present paper we go beyond previous works on UPB by demonstrating a reliable protocol that allows to operate any such system under pulsed excitation. In fact, this can be technically achieved by a combination of excitation pulse tailoring and post-selective temporal filtering of the output stream to purify the statistics of the emitted radiation, similarly to what has been already demonstrated for quantum dot-based single photon sources^13^. The latter achievement allows to overcome a previously believed limitation, and provides a scheme to devise a true single photon source out of a generator of antibunched radiation. The efficiency of such a single photon source in realistic devices is analyzed in detail, which is shown to outperform the best heralded sources demonstrated so far in key figures of merit, especially operation power and spatial footprint.

## Results

**Figure 1:**
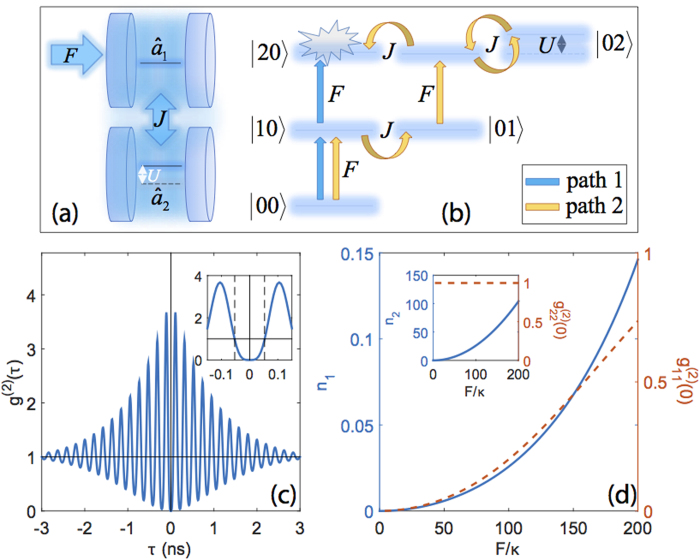
Under cw excitation ? (**a**) Schematic representation of an asymmetrically driven photonic molecule. Each cavity is characterized by a single resonant mode in the spectral region of interest, and only the first cavity is driven by an external coherent field. (**b**) The corresponding ladder scheme of the lowest few energy levels, associated to photon occupation number states in the two cavities. (**c**) The computed second-order correlation function of the quantum field in the first cavity, *g*^(2)^(τ), plotted as a function of time delay. Inset: a magnification of the photon antibunching region close to zero delay. (**d**) Average photon occupation of the first cavity (*n*_1_, full line), and corresponding value of *g*^(2)^(0) (dashed line), computed as a function of the cw driving field amplitude. Inset: Same quantities plotted for the second cavity.

Following Refs. 20 and 21, we consider the UBP in a system of two tunnel-coupled cavities, i.e. a *photonic molecule*, as sketched in Fig. 1a. The quantum model of UPB has been thoroughly characterized^22^, and it is briefly summarized in the Methods section. The relevant physical parameters are the tunnel coupling rate between the two cavities (*J*/*ħ*), the driving rate on the first cavity (*F*/*ħ*), the driving frequency (*ω*_*L*_), the effective photon-photon interaction energy in each cavity (*U*_*j*_, *j* = 1, 2), which originates from the intrinsic material *χ*^(3)^^19^, and the cavities loss rates *κ*_*j*_. Detrimental pure-dephasing processes are known to be negligible if the overall dephasing rate is much smaller than *U*_*j*_/*ħ*^22^. For a photonic crystal cavity in silicon, this condition is largely fulfilled^26^. Finally, the model can be generalized to include input and output quantum channels^27^. We will assume *ω*_*j*_ _*=*_ *ω*_*c*_, *U*_*j*_ _*=*_ *U* and *κ*_*j*_ = *κ* in the following, without loss of generality.

A scheme of the lowest 6 levels on the basis of photon-number states, 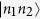, is given in Fig. 1b. The different excitation pathways leading from the initial ground state to the state 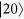
—corresponding to two-photon occupation of the first (driven) cavity—are highlighted. The UPB is essentially based on suppression of such double occupation by a careful tuning of the model parameters, leading to destructive quantum interference between the two alternative pathways. The optimal UPB conditions^21^ are given by 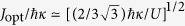, and 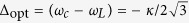, and will be assumed to hold in what follows.

We consider the UPB mechanism in a SOI nanophotonic platform, where the cavity-field confinement in a diffraction-limited mode volume, *V* ~ (*λ*/*n*)^3^, enhances the effective photon-photon interaction, *U*. A realistic order of magnitude estimate in a crystalline silicon photonic crystal nanocavity leads to 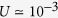 *μ*eV (see also Supplementary Information)^22^. Assuming a quality factor 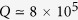
—now routinely achieved at telecom wavelengths (i.e., 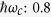 eV)^23^,^28^,^29^—we set 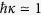 *μ*eV, and hence *U*/*ħκ* *=* 0.001. To fulfill the optimal UPB conditions, the remaining parameters take values Δ_opt_ = −0.29*κ* and *J* = 19.6*ħκ*, respectively.

The steady state results under cw driving are summarized in Fig. c and d. The time-dependent normalized second-order correlation function, 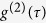 (see Methods section) is considered as the reference figure of merit for single-photon blockade^30^,^31^,^32^ and plotted in Fig. 1c. A strong antibunching is present over a time-delay window τ<100 ps. At longer delays, strong oscillations are present on the timescale *h*/*J*, arising from the interferential nature of the UPB mechanism^21^. The average photon occupations in the two cavities, 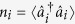, and the corresponding zero-delay correlation, g^(2)^(0), are displayed as a function of the driving field amplitude, *F*, in Fig. 1d: UPB occurs at low average occupation of the driven cavity, while the occupation of the non-detected cavity is much larger (see inset). This figure of merit is relevant to determine the maximal single-photon emission rate that can be achieved in such a device under cw pumping, given by *R*_em_ = *n*_1_*κ*/2π. As a*n* example, for 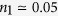 (corresponding to *F/ħκ* *~* 30 and g^(2)^(0) < 0.5), *R*_em_ > 10 MHz can be expected, with an input power as low as 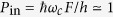 nW. In fact, the optimal UPB relation between *U* and *J* leads to a condition (without numerical pre-factors, for convenience) 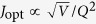; this means that the required input-power in UPB scales down roughly as 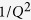, i.e. the larger the cavity Q, the smaller *J*_opt_ can be to have antibunching by keeping the average number of photons in the first cavity less than 0.1 (according to Fig. 1d). The same figure of merit simultaneously allows to increase the antibunching time window, scaling as 1/*J*_opt_ (see Fig. 1c).

Single-photon sources on demand require the emission of single-photon pulses at deterministic times. However, in the UPB mechanism the emitted light is sub-Poissonian only within a time delay shorter than *h*/*J*, as shown in Fig. 1c. For short input pulses, the outgoing pulses will last at least as long as the cavity lifetime. Therefore, the condition 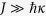 would apparently prevent the device from operating in a pulsed regime^21^.

**Figure 2:**
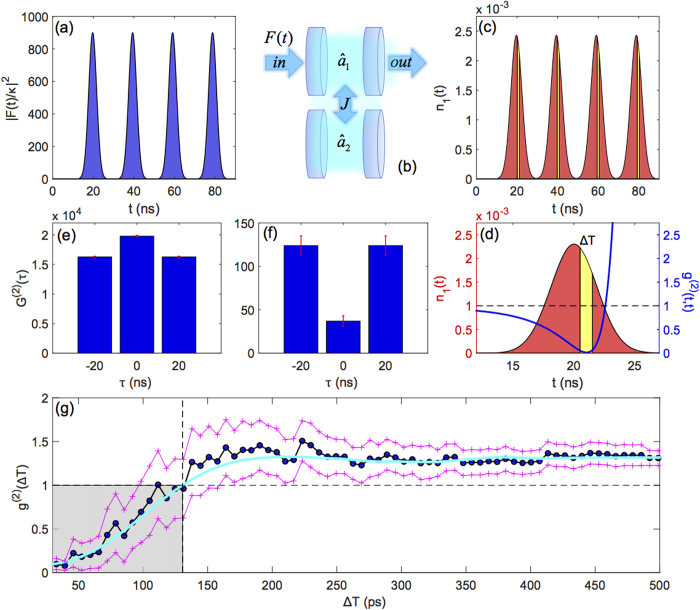
Unconventional photon blockade under pulsed excitation. (**a**) Input pulse sequence driving the first cavity, as schematically represented in (**b**). Pulse duration is set to *σ* _t_= 4 ns at 50 MHz repetition rate. (**c**) Corresponding cavity output, i.e. average population in the driven cavity as a function of time. The filtering window is schematically superimposed within each pulse (yellow areas). (**d**) Detail of a single pulse from the output sequence in panel (**c**). The blue curve shows the equal time second-order correlation function, *g*^(2)^(*t*,*t*) (scale on the right). (**e**) Un-normalized and un-filtered second-order correlation over the whole pulse sequence (see Supplementary Information). (**f**) Same, after time-filtering the pulses with Δ*T* *=* 75 ps. (**g**) Full Montecarlo wave function simulation of the time-filtered second-order correlation function under pulsed excitation, as a function of the width Δ*T* of the filtering window (disks), and relative error span (crosses). The cyan line is the two-time second-order correlation calculated by solving the quantum master equation (see Supplementary Information). The model parameters assumed in these simulations are the same as in Fig. 1.

Here we show for the first time that UPB under pulsed excitation is possible by exploiting temporal filtering of the output signal. In Fig. 2, the results of a numerical experiment are reported for the UPB device considered in the previous section, where a train of gaussian pulses drives the first cavity (Fig. 2a–c). A sequence of outgoing pulses from cavity 1 is modeled by solving the quantum master equation (see Supplementary Information for details), and shown in Fig. 2c. Focusing on a single outgoing pulse, the equal-time second-order correlation is plotted in Fig. 2d (blue curve), where a well-defined time window clearly exists—within the pulse emitted from a UPB device—during which light is antibunched over a time delay shorter than *h*/*J*.

As a consequence, pulsed operation can be achieved by gating the outgoing pulses in time, in order to retain only a timeframe of duration <*h*/*J*. In practice, this could be achieved with an integrated all-optical switch triggered by the input pulse, as it was already shown experimentally^13^. The second-order correlation function under pulsed excitation (see Supplementary Information) is shown in Fig. 2e–g. The histograms in Fig. 2e,f show the un-normalized correlation signal, 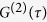, integrated over the whole pulse sequence in (Fig. 2e) and in the presence of filtering with a time window ΔT = 75 ps (Fig. 2f), respectively. They reveal a strong reduction of the two photon counts within a pulse after filtering, which is a key result of this paper. Figure. 2g displays the dependence of the filtered second-order correlation versus the filtering time window, Δ*T*. The Montecarlo data (blue disks), directly obtained from the photon count statistics (see Supplementary Information), are reproduced by a master equation treatment (cyan curve), which confirms the reliability of this result. Photon antibunching (gray area) is achieved below Δ*T* = 130 ps, while the single photon regime—requiring the condition *g*^(2)^(Δ*T*) < 0.5 – is obtained for Δ*T* *<* 90 ps. When assuming 5×10^7^ pulses per second and a peak value *F* ~ 150*ħ*κ, after the temporal filtering the Montecarlo data indicate a single-photon yield at a rate of about 0.45 MHz. Under these conditions, the driven cavity reaches a peak value of the average photon occupation *n*_1_ ~ 0.075, close to the largest occupancy for which UPB is expected according to Fig. 1c [i.e., *g*^(2)^(0) < 0.5]. Remarkably, this peak value of *F*(*t*) implies an intracavity energy of less than 10^−2^ fJ per pulse. This corresponds to an input energy that can be quantified as ~0.5 fJ per pulse, according to typical excitation schemes of photonic crystal integrated circuits^25^. We notice that this is extremely low when compared to the state-of-art parametric sources demonstrated so far in integrated silicon-based platforms and based on four-wave mixing and heralding (typical input powers in the 100 mW range), for a comparable output rate in the few 100 kHz range^14^,^15^,^16^.

**Figure 3:**
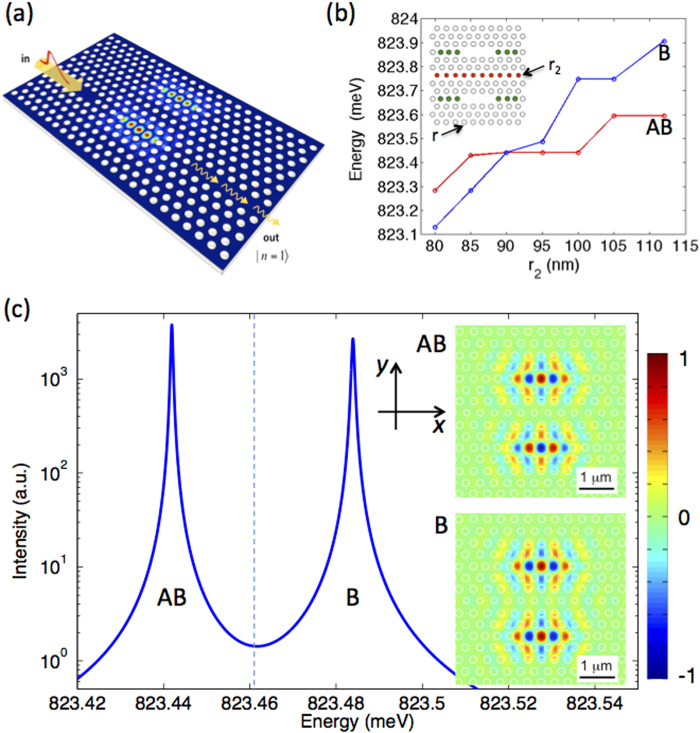
Realization of a SOI integrated single-photon source. (**a**) Artistic view of an integrated SOI photonic crystal chip realizing input/output channels and UPB through a photonic crystal molecule. (**b**) Fine-tuning of the normal mode splitting of a L3 photonic crystal molecule (see text) through variation of the radius (*r*_2_) in the middle row of holes (red-highlighted in the inset). The holes highlighted in green are shifted off from the cavities center to optimize the Q-factor^23^,^24^. The hole radius of the surrounding photonic crystal lattice is *r* = 112 nm. All the design parameters are given in detail in the Supplementary Information associated to this manuscript. (**c**) Spectrum of the photonic crystal molecule designed to have the parameters corresponding to the results shown in Figs 1 and 2. The two normal mode resonances are indicated as bonding (B) and antibonding (AB), respectively. The exciting laser frequency at the optimal UPB condition is schematically indicated (vertical dashed line). Inset: *E*_*y*_ component plotted for the two normal modes, superimposed to the photonic crystal design showing the footprint area of the coupled cavities device. The reference directions *x* and *y* are explicitly indicated.

A feasible realization is hereby proposed in an integrated SOI photonic crystal platform. These structures benefit from a remarkably advanced and well established fabrication technology, with nanocavities having recently achieved 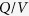-values well above the UPB requirements^23^,^25^,^28^,^29^. As a schematic example, we show in Fig. 3a a double-cavity device in a photonic crystal circuit. This configuration allows to selectively drive one cavity from the input waveguide channel, and simultaneously collect part of the light emitted from the same cavity into the output waveguide (the remaining part being emitted through out-of-plane losses). As an elementary building block, we consider a L3 photonic crystal cavity in a thin silicon membrane, designed for operation at the preferred telecom wavelength, *λ* = 1.5 *μ*m (~0.825 eV). The cavity consists of three missing air holes in a triangular air-hole lattice. This cavity was recently optimized to show a measured quality factor regularly exceeding one million^23^,^24^. We use here a L3 cavity design with theoretical unloaded (i.e., valid for the isolated cavity) 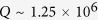 (see Supplementary Information for details on the structure parameters, such as hole radius and lattice constant). When coupling to the access waveguides, the loaded Q-factor can be engineered in the range 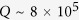, as we have verified by 3D finite-difference time-domain simulations (3D-FDTD, not shown). From the calculated mode profile, the effective nonlinearity for this device is estimated (see Supplementary Information) in the range *U* ≃ 0.8×10^−3^ *μ*eV, close to what was assumed in the model calculations above.

The photonic crystal molecule can be obtained by vertically aligning two L3 cavities, separated by 5 rows of holes (i.e., 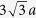 center-to-center)^33^. The hole radius in the central row, *r*_2_, can be varied to fine tune the normal modes splitting at the desired value^34^, i.e. Δ = 2*J* according to Eq. 1 in Methods. In Fig. 3b we show such a simulated fine tuning by 3D-FDTD calculations. For *r*_2_ ~ 95 nm, the normal mode splitting between the two resonances, identified as bonding (B) and antibonding (AB) according the the spatial profile of the *E*_*y*_ component, is calculated as Δ ≃ 44 *μ*eV, i.e. remarkably close to the condition *J*/*ħκ* *=* 19.6 assumed in the previous calculations, when we consider the loaded value *ħκ* ≃ 1 *μ*eV. We notice that similar values and dynamic control of the normal mode splitting have been already shown experimentally in SOI photonic crystal platforms operating in a very similar wavelength range^35^. The spectrum for such an optimal structure is shown in Fig. 3c. The two resonances have unbalanced Q-factors of ~1.1×10^6^ (AB) and ~1.3×10^6^ (B), respectively, which can also be exploited to enhance the degree of antibunching in UPB^22^.

Before concluding, we discuss how to circumvent the most relevant and potentially detrimental effects for the realization of UPB in a SOI platform. First of all, two-photon absorption (TPA), related to the imaginary part of the silicon *χ*^(3)^, is also enhanced by confinement in the L3 cavities. However, a quantitative estimate of this contribution has been given in Ref. 19 by which we can infer a TPA loss rate that is on the order of *κ*_*TPA*_/*κ* *<* 10^−2^ for the present case, also considering the low input powers necessary to achieve UPB. Second, thermal effects can give rise to pure dephasing of the cavity resonances, which depends on optomechanical coupling with the background phonons. For the L3-type silicon photonic crystal cavities considered here, this contribution has been quantitatively estimated and shown to be negligible even at room temperature (i.e., a pure dephasing rate γ^*^/*κ* *~* 10^−7^)^26^. Finally, unavoidable fabrication imperfections should be corrected by device post-fabrication processing. In particular, fine and selective cavity tuning has been already shown for photonic crystal cavities with different techniques, even in the presence of very large Q-factors^35^,^36^,^37^.

## Discussion

We have theoretically shown that an integrated nanophotonic platform based on CMOS-compatible SOI technology can be engineered to achieve single photon emission by an unconventional photon blockade mechanism. Besides opening the way to the first experimental demonstration of the UBP, our results show that such unconventional mechanism allows for pulsed excitation, which represents a key ingredient for a useful source where each pulse potentially triggers emission of a single photon.

Such an alternative single-photon source could be characterized by a very low input power operation, i.e. comparable to standard single-photon devices based on cavity QED but much lower than typical integrated single-photon sources based on enhanced four-wave mixing and heralding. Moreover, this is achieved by an unprecedented small footprint area, significantly smaller than recently realized heralded sources in integrated SOI chips. In fact, notice that the footprint of this prospected device is essentially given by the spatial extension of the photonic crystal molecule and the necessary lattice around it. For the structure simulated in Fig. 3, we estimate a minimal footprint area on the order of a few *μ*m^2^ (see, e.g., the inset in Fig. 3c). In practice, this is significantly smaller than current heralded sources fabricated with the same SOI technology and based on coupled resonator optical waveguides^14^ or spatially multiplexed photonic crystal waveguides^17^. We also stress the generality of the proposed scheme, which could be extended to other types of nonlinearities^38^, and could eventually lead to the realization of novel quantum devices^39^,^40^ for applications in integrated quantum metrology and logic.

In summary, by combining an extremely low input power, a small footprint area, and no quantum emitter required for single-photon generation, such a device might have significant impact on the development of integrated silicon quantum photonics, by introducing a new concept in the generation of pure quantum states of light at arbitrary wavelengths (e.g., in the telecom band), that is fully compatible with current semiconductor technology, working at room temperature, and a viable alternative to single-photon nonlinear devices based on cavity-QED with artificial atoms or single atomic-like emitters that are presently lacking in SOI integrated platforms.

## Methods

### Model Hamiltonian

The second quantized hamiltonian of the driven nonlinear photonic molecule is expressed (to leading linear and nonlinear orders) as^20^,^21^,^22^


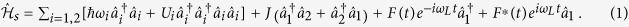


The first terms in Eq. 1 describe two harmonic oscillators, *J*/*ħ* is the tunnel coupling rate between the two resonators, *F*(*t*)/*ħ* is the coherent pumping rate on the first cavity at the laser frequency ω_*L*_, and the photon-photon interaction energy in each resonator is related to the material *χ*^(3)^^19^,^22^. A description of this quantity and an estimation for the photonic crystal cavities considered here are given in the Supplementary Information. Starting from this Hamiltonian, the various time-dependent photon correlation functions for light collected after cavity 1, generally defined as


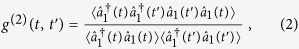


were numerically simulated by using both the Montecarlo wave function method and by directly solving the master equation for the density matrix (see details in Supplementary Information). In both cases, the numerical solutions were computed on a truncated Hilbert space of dimensions (*N*_1_×*N*_2_)^2^, where *N*_1_ = 4 and *N*_2_ = 18 are the maximum photon occupations allowed in cavities 1 and 2, respectively. While the master equation results were obtained from a modern workstation embedding 16 Gb of RAM, the Montecarlo data were produced by 10 nodes of 16 cores and 32 Gb RAM memory each, run on a high-end cluster for a few weeks of continuous computational time.

## Additional Information

**How to cite this article**: Flayac, H. *et al.* An all-silicon single-photon source by unconventional photon blockade. *Sci. Rep.* **5**, 11223; doi: 10.1038/srep11223 (2015).

## Supplementary Material

Supplementary Information
