# Supplementary material for: An all-silicon single-photon source by unconventional photon blockade
Source: Sci Rep. 2015 Jun 10;5:11223. doi: 10.1038/srep11223 (PMC4462143; doi:10.1038/srep11223)
Supplement: Supplementary Information [file srep11223-s1.pdf]

Supplementary Information to
"An all-silicon single-photon source by unconventional photon blockade"

Hugo Flayac,[1] Dario Gerace,[2] and Vincenzo Savona[1]

[1] *Institute of Theoretical Physics, École Polytechnique Fédérale de Lausanne EPFL, CH-1015 Lausanne, Switzerland*
[2] *Department of Physics, University of Pavia, I-27100 Pavia, Italy*

We detail here the Montecarlo and master equation treatments used to produce the results of figures 1 and 2 in the main text. We also give details on the photonic crystal cavities design, and the corresponding estimation of the single-photon nonlinearity reported in the manuscript.

I. MONTECARLO WAVE FUNCTION METHOD

The statistics of the photons emitted by the system under pulsed excitation were first addressed by performing quantum Montecarlo simulations [1]. This method not only allows to work with larger truncated Hilbert spaces but also provides direct access to individual photon counts, thus embodying the closest theoretical simulation of an actual Hanbury Brown-Twiss experiment. In brief, the algorithm is based on the stochastic evolution of the system wave function through the Schrödinger equation

$$\hat{\mathcal{H}}\left|\psi\right\rangle = i\hbar\frac{\partial}{\partial t}\left|\psi\right\rangle , \quad (1)$$

written for the non-Hermitian effective Hamiltonian

$$\hat{\mathcal{H}} = \hat{\mathcal{H}}_s - \frac{i\hbar}{2}\sum_j \kappa_j \hat{a}_j^\dagger \hat{a}_j , \quad (2)$$

The non Hermitian part of 2 results in a decay of the norm $\langle\psi \mid \psi\rangle$. During the evolution of Eq. 1, random numbers $0 < r < 1$ are drawn and the condition $\langle\psi \mid \psi\rangle \leq r$ decides for the action of a jump operator, $\hat{a}_j\left|\psi\right\rangle$, corresponding to the measurement of a photon. The proper quantum jump operator is chosen such that j is the smallest integer satisfying $\sum_j P_j \geq r$, where P_j are the probabilities for the mode j to emit a photon at a given time. Each evolution of Eq. 1 produces a stochastic quantum trajectory associated with the state $\left|\psi(t)\right\rangle_j$, and the procedure can be repeated N times to form an ensemble average of realizations in view of approximating the system density matrix as $\hat{\rho}\left(t\right) \underset{N\to\infty}{=} \left|\Psi\left(t\right)\right\rangle\left\langle\Psi\left(t\right)\right|$, where

$$\left|\Psi\left(t\right)\right\rangle = \sum_{j=1}^{N}\left|\psi\left(t\right)\right\rangle_j / N , \quad (3)$$

and its potential mixed nature. Any observable or correlation are obtained from $\langle\hat{O}(t)\rangle = \mathrm{Tr}[\hat{\rho}(t)\hat{O}]$. The full access to photon counts and emission times history allows to mimic the experimental detection scheme. The two-times second-order correlation function, $g^{(2)}(t_1, t_2)$, can be reconstructed from the statistics of photons delays analogously to a Hanbury Brown and Twiss (HBT) setup. Further details on the numerical procedure employed to obtain the results of Fig. 2 in the main text are given in the following.

II. TWO-TIME CORRELATIONS UNDER PULSED EXCITATION

In our Montecarlo simulations we have worked on the basis of trajectories containing single pulses, which makes the data analysis more flexible. We have tracked the quantum jumps performed by the driven cavity from which the photon antibunching is expected. We point out that in reality one should expect a weak mixing between both cavity fields to occur in the guiding channels. It can be accounted for within an input-output treatment. In such a case the laser detuning should be properly adapted according to the prescriptions of Ref. 2. To analyze delays within a given pulse we need to track the trajectories where *at least* two quantum jumps occurred within ΔT and these events are obviously rare given the relatively small occupation of the cavity 1 as one can see from Fig. 2 of the main text. We have therefore performed a large campaign of massively parallelized simulations on an high end cluster based on $N = 1.5 \times 10^8$ pulses from which we have recorded the whole quantum jump history. We considered pulses of duration 4 ns separated by 20 ns to avoid any overlap bringing some unwanted pulse to pulse correlations. Our simulation therefore covers not less than 1 seconds of recorded data.

To build the Monte-Carlo curve of Fig. 2g (blue disks), we have worked on quantum jumps that occurred in a time window of width $\Delta T = T_2 - T_1 = 1.57$ ns centered on the $g^{(2)}(t, t)$ minimum (see yellow surface and blue curve of Fig. 2f) mimicking the temporal filtering. Inside this global window we have considered sub-windows of variable duration $\Delta t = t_2 - t_1$ ranging from 6.5 ps to ΔT. Each of these sub-windows was gradually displaced by Δt within ΔT, starting from the condition $t_1 = T_1$ ($t_2 = t_1 + \Delta t$) and until $t_2 = T_2$ ($t_1 = t_2 - \Delta t$) is fulfilled. For a given value of Δt, the un-normalized second order correlation $G^{(2)}(\Delta t)$ is obtained from the sum of photon pair counts recorded by slicing the time window, which increases the statistics by $N_w = \Delta T/\Delta t$. Therefore it allows to work with a number of counts that would correspond to $N \times N_w$ trajectories (pulses) reducing by N_w the required computational time. Finally, the $g^{(2)}(\Delta t)$ is obtained by normalizing $G^{(2)}(\Delta t)$ to the number double counts expected from a Poissonian statistics. The errors (magenta curve) are computed from the square root of the number of counts averaged over the sliding windows. Obviously the error is inhomogeneous versus Δt, given

that N_w is variable, and it is small both in the regions of narrow and wide Δt where respectively N_w is large and number of counts is important. The previously described procedure is summarized in Fig. S1 (see captions).

To build the histograms of Fig. 2d-e displaying the pulse-to-pulse statistics, we have performed a Montecarlo rearrangement of our single pulse trajectories to randomize their time ordering, as it would be obtained from many pulse trajectories or in an actual experimental situation.

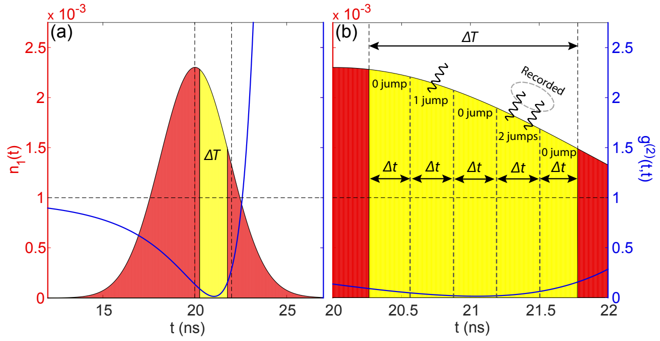

FIG. 1. (a) Average occupation of cavity 1 (red) and the equal time second order correlation function (blue line). The yellow region highlights the global time window ΔT from which the photon counts are extracted. (b) Zoom in between the vertical dashed lines of panel (a) showing an illustrative set of sub-windows for a given value of Δt. The wavy lines illustrate and example of quantum jump series for a given trajectory. Only the 2 jumps event are kept for the $G^{(2)}(\Delta t)$ statistics. 3 and more jumps events are totally absent in the conditions we consider.

III. QUANTUM MASTER EQUATION

The master equation for the density matrix reads

$$\dot{\rho} = \frac{1}{i\hbar}[\rho, \hat{\mathcal{H}}_s] + \mathcal{L}^{(1,2)} \,, \tag{4}$$

where losses are accounted for through Liouvillian operators in the usual Lindblad form for the two resonators modes, $\mathcal{L}^{(1,2)} = \sum_{i=1,2} \kappa_i [\hat{a}_i \rho \hat{a}_i^\dagger - 0.5(\hat{a}_i^\dagger \hat{a}_i \rho - \rho \hat{a}_i^\dagger \hat{a}_i)]$. Further sources of loss, such as nonlinear absorption (e.g. related to the imaginary part of $\chi^{(3)}$) or pure dephasing, could also be added to Eq. 4 (see, e.g. 5 and 13), but we neglect them here for simplicity. Moreover, the model can be generalized to include input and output quantum channels [2].

Single-time evolution and steady state numerical results of Eq. 4 can be straightforwardly performed, as in Refs. 3–5. Here, we were additionally able to confirm the Montecarlo results (cyan curve in Fig. 2g of the main text). The un-normalized and normalized two-times second-order correlation functions of cavity 1 were computed as

$$G^{(2)}(t, t') = \mathrm{Tr}\left[\hat{a}_1^\dagger \hat{a}_1 U_{t\to t'}\left(\hat{a}_1^\dagger \hat{a}_1 \hat{\rho}(t')\right)\right] \tag{5}$$

$$g^{(2)}(t, t') = \frac{G^{(2)}(t, t')}{\mathrm{Tr}\left[\hat{a}_1^\dagger \hat{a}_1 \hat{\rho}(t)\right] \mathrm{Tr}\left[\hat{a}_1^\dagger \hat{a}_1 \hat{\rho}(t')\right]} \,, \tag{6}$$

where $\hat{\mathcal{U}}_{t\to t'}(\hat{O})$ is the propagator of the operator $\hat{O}$ from t to t' associated with Eq. 4. The photon statistics produced within a time window $\Delta t = t_2 - t_1$ is obtained from

$$g^{(2)}(\Delta t) = \frac{\iint_{\Delta t} G^{(2)}(t, t')\, dt dt'}{\iint_{\Delta t} n_1(t) n_1(t') dt dt'} \tag{7}$$

where $n_1(t) = \mathrm{Tr}[\hat{a}_1^\dagger \hat{a}_1 \hat{\rho}(t)]$. This exact calculation perfectly reproduces the Montecarlo wave function results within the error envelope, as it is reported in Fig. 2g (cyan curve).

IV. PHOTONIC CRYSTAL CAVITIES DESIGN

Photonic crystal cavities allow to achieve record figures of merit today, such as ultra-small mode volumes and ultra-high quality factors [6]. One of the most used photonic crystal cavity designs is realized by removing three air holes in a triangular lattice [7], which is usually defined a L3 point defect. Recently, a combination of fast simulation tools [8] and genetic optimization [9] have allowed to show that Q factors largely exceeding 10^6 can be designed for such cavities, which was shown experimentally [10].

For the photonic crystal cavities design used in this work, we started from a standard SOI photonic crystal membrane, with the silicon layer thickness of 220 nm. We set the lattice constant to $a = 400$ nm and the holes radius to $r = 112$ nm ($r/a = 0.28$) to tune the cavity mode resonant wavelength in the relevant telecom band, i.e. $\lambda = 1.5$ μm (~ 0.825 eV). The three holes along the cavity axis have been shifted by $s_1 = 120$ nm ($s_1/a = 0.3$) $s_2 = 100$ nm ($s_2/a = 0.25$), and $s_3 = 40$ nm ($s_3/a = 0.1$), to reach a theoretical (unloaded) $Q \sim 1.25 \times 10^6$. Since we aim at coupling these cavities with access and output waveguides, we thus allow the loaded Q-factor to be in the 0.8×10^6 range.

A plot of the normalized electric field intensity, i.e. .the function $|\vec{\alpha}(\mathbf{r})|^2$ with $\int |\vec{\alpha}(\mathbf{r})|^2 \mathrm{d}\mathbf{r} = 1$, is shown in Fig. S2 for our optimized L3 cavity design, which was the building block for the the photonic crystal molecules in Fig. 3 of the main text.

V. ESTIMATING THE EFFECTIVE PHOTON-PHOTON INTERACTION

In this work, we focus on photonic nanostructures in silicon, which is a strongly nonlinear material already at

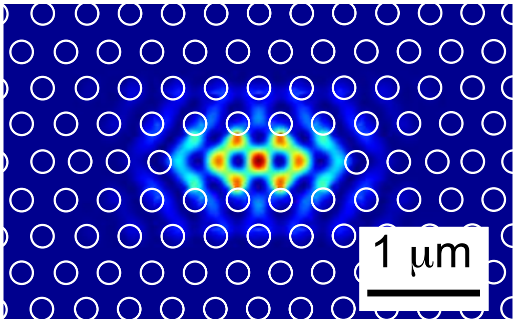

FIG. 2. Electric field intensity profile at the center of the silicon photonic crystal membrane.

the level of classical electromagnetic response. In particular, bulk silicon is characterized by a relatively large $\chi^{(3)}$ susceptibility, while nominally $\chi^{(2)} = 0$ (neglecting surface contributions) owing to the centrosymmetric nature of the elementary crystalline cell [11]. Strongly enhanced nonlinear effects have been already reported in L3 photonic crystal cavities [12].

The photon-photon interaction energy in each resonator is given in terms of the material $\chi^{(3)}$ by the simplified expression [13]

$$U = \frac{D(\hbar\omega_i)^2}{8\varepsilon_0} \int \mathrm{d}\mathbf{r}\, \frac{\chi^{(3)}(\mathbf{r})}{\varepsilon^2(\mathbf{r})} \left|\alpha(\mathbf{r})\right|^4 , \qquad (8)$$

where $\vec{\alpha}(\mathbf{r})$ is the three-dimensional cavity field profile, normalized as $\int |\vec{\alpha}(\mathbf{r})|^2 \mathrm{d}\mathbf{r} = 1$, and D represents the multiple contributions of the same order of magnitude given by the different elements of the $\chi^{(3)}$ tensor [11].

From the calculated mode profile shown in Fig. S2, the effective nonlinearity of such a silicon nanocavity can be estimated through Eq. 8, by using $\chi^{(3)} \sim 0.9 \times 10^{-18}$ $\mathrm{m}^2/\mathrm{V}^2$, which is an appropriate order of magnitude for the elements of the bulk silicon third-order susceptibility tensor [11], and D=24 [14]. For the cavity mode profile of our optimized photonics crystal structure, see Fig. S2, a quantitative estimate of this integral results in $U \simeq 0.8 \times 10^{-3}$ μeV, close to what was assumed in the model calculations of the main text and confirming the order of magnitude estimates already given in the literature [5].

[1] R. Dum, P. Zoller, and H. Ritsch, "Monte Carlo simulation of the atomic master equation for spontaneous emission." Physical Review A **45**, 4879 (1992).

[2] H. Flayac and V. Savona. "Input-output theory of the unconventional photon blockade." Phys. Rev. A **88**, 033836 (2013).

[3] T. C. H. Liew and V. Savona. "Single photons from coupled quantum modes." Phys. Rev. Lett. **104**, 183601 (2010).

[4] M. Bamba, A. Imamoğlu, I. Carusotto, and C. Ciuti. "Origin of strong photon antibunching in weakly nonlinear photonic molecules." Phys. Rev. A **83**, 021802(R) (2011).

[5] S. Ferretti, V. Savona, and D. Gerace. "Optimal antibunching in passive photonic devices based on coupled nonlinear resonators." New J. Phys. **15**, 025012 (2013).

[6] M. Notomi. "Manipulating light with strongly modulated photonic crystals." Rep. Prog. Phys. **73**, 096501 (2010).

[7] T. Akahane, T. Asano, B.-S. Song, and S. Noda. "High-Q photonic nanocavity in a two-dimensional photonic crystal." Nature **425**, 944 (2003).

[8] L. C. Andreani and D. Gerace. "Photonic crystal slabs with a triangular lattice of triangular holes investigated using a guided-mode expansion method." Phys. Rev. B **73**, 235114 (2006).

[9] M. Minkov and V. Savona. "Automated optimization of photonic crystal slab cavities." Sci. Rep. **4**, 5124 (2014).

[10] Y. Lai, S. Pirotta, G. Urbinati, D. Gerace, M. Minkov, V. Savona, A. Badolato, and M. Galli. "Genetically designed L3 photonic crystal nanocavities with measured quality factor exceeding one million." Appl. Phys. Lett., **104**, 241101 (2014).

[11] R. W. Boyd, *Nonlinear Optics* (Academic Press, 2008).

[12] M. Galli, D. Gerace, K. Welna, T. F. Krauss, L. O'Faolain, G. Guizzetti, and L. C. Andreani. "Low-power continuous-wave generation of visible harmonics in silicon photonic crystal nanocavities." Opt. Express **18**, 26613 (2010).

[13] S. Ferretti and D. Gerace. "Single-photon nonlinear optics with Kerr-type nanostructured materials." Phys. Rev. B **85**, 033303 (2012).

[14] J. E. Sipe, D. J. Moss, and H. M. van Driel. "Phenomenological theory of optical second- and third-harmonic generation from cubic centrosymmetric crystals," Phys. Rev. B **35**, 1129 (1987).
